# Using novel oxidative phosphorylation inhibitors to attenuate drug resistance in human gliomas

**DOI:** 10.17179/excli2025-8193

**Published:** 2025-03-12

**Authors:** Chia-Kuang Tsai, Chin-Yu Lin, Yung-Lung Chang, Fu-Chi Yang, Chung-Hsing Chou, Yu-Chian Huang, Dueng-Yuan Hueng

**Affiliations:** 1 Department of Neurology, Tri-Service General Hospital, National Defense Medical Center, Taipei 11490, Taiwan; 2 Department of Biochemistry, National Defense Medical Center, Taipei 11490, Taiwan; 3 Department of Neurological Surgery, Tri-Service General Hospital, National Defense Medical Center, Taipei, 11490, Taiwan

**Keywords:** glioblastoma, Gboxin, oxidative phosphorylation capacity, PLK2

## Abstract

Glioblastoma multiforme (GBM) is an aggressive brain tumor with a poor prognosis, worsened by resistance to temozolomide (TMZ). TMZ-induced DNA damage is counteracted by the repair enzyme O-6-methylguanine-DNA methyltransferase (MGMT), promoting tumor recurrence. Targeting oxidative phosphorylation (OXPHOS), essential for cellular energy production, offers a potential therapeutic strategy to overcome TMZ resistance and improve GBM treatment outcomes. Gboxin, a small-molecule drug, selectively inhibits OXPHOS by targeting complex V, with minimal toxicity to normal cells. It accumulates in the mitochondria of GBM cells, exploiting their high membrane potential and pH, thereby inhibiting cell proliferation. This study evaluates Gboxin's efficacy in TMZ-resistant (TMZ-R) GBM. Results show that Gboxin suppresses the growth of both TMZ-sensitive and TMZ-R GBM cells by inhibiting proliferation, inducing apoptosis, and reducing OXPHOS activity. These findings were confirmed in an *in vivo* model, highlighting Gboxin as a promising therapeutic for both TMZ-sensitive and TMZ-R GBM.

See also the graphical abstract(Fig. 1).

## Abbreviations

CDK: cell cycle dependent kinase

CGGA: Chinese Glioma Genome Atlas 

DMEM: Dulbecco’s modified Eagle’s medium

DMSO: Dimethyl sulfoxide

FACS: Fluorescence activated cell sorting

FBS: fetal bovine serum

FCCP: Carbonyl cyanide 4-(trifluoromethoxy)phenylhydrazone

GBM: Glioblastoma multiforme

GENT2: Gene Expression Database of Normal and Tumor Tissues 2

i.p.: intraperitoneal injection

MGMT: O-6-methylguanine-DNA methyltransferase

OCR: oxygen consumption rate

OXPHOS: Oxidative phosphorylation

PI: propidium iodide

PLK2: polo-like kinase 2

RIPA: Radioimmunoprecipitation assay buffer

scRNA-Seq: single-cell RNA sequencing

SDs: standard deviations

TCA: tricarboxylic acid

TMZ: temozolomide

## Introduction

Glioblastoma multiforme (GBM) is an aggressive brain tumor with a poor survival time of less than 2 years, mainly because of its invasive nature, which limits the extent of surgical resection and contributes to the high recurrence rate (Marenco-Hillembrand et al., 2020[10]; Ostrom et al., 2017[13]; Wen and Kesari, 2008[26]). GBM cells migrate to and invade surrounding brain tissue, and this process is driven by various molecular factors. Moreover, the occurrence of resistance to temozolomide (TMZ), the main chemotherapy used for GBM, is a significant factor that further worsens treatment outcomes (Hegi et al., 2005[8]). TMZ works by stimulating cell death in gliomas through damage to DNA via methylation, especially at the N-7 or O-6 positions of guanine residues. Nevertheless, GBM cells counteract this damage by upregulating a DNA repair enzyme called O-6-methylguanine-DNA methyltransferase (MGMT). This leads to the formation of TMZ-resistant (TMZ-R) cells, which are crucial for tumor recurrence (Hegi et al., 2005[8]). Thus, strategies to counteract TMZ resistance and improve survival outcomes are urgently needed.

Oxidative phosphorylation (OXPHOS) is crucial for cellular energy production and involves more than 90 proteins encoded in the nucleus and mitochondria (Tang et al., 2020[22]). This complex process includes four electron transport chain complexes (I to IV) that convey electrons from the tricarboxylic acid (TCA) cycle and fatty acid oxidation to oxygen. A proton gradient is then created across the inner mitochondrial membrane, which urges complex V (F0F1 ATP synthase) to generate ATP (Nolfi-Donegan et al., 2020[12]). Modifying mitochondrial activity by targeting OXPHOS has potential value in GBM treatment (Shen et al., 2015[17]; Wurth et al., 2013[27]; Garcia et al., 2021[6]).

The cell cycle is governed by various CDKs that bind to their respective cyclin partners to form complexes. In proliferating mammalian cells, ATP is produced primarily through OXPHOS, which involves a series of five multisubunit complexes that facilitate electron transport and energy generation (Arnold and Finley, 2023[2]). Research indicates a tight connection between CDKs, mitochondrial respiration and cell cycle regulation (Taguchi et al., 2007[21]). The G1/S and G2/M checkpoints are sensitive to energy levels and require sufficient bioenergetic resources for the synthesis of the necessary biomolecules for cell cycle progression (Sweet and Singh, 1999[20]). 

Gboxin, a small-molecule drug created and published in 2019, exclusively targets OXPHOS by impeding complex V (Shi et al., 2019[18]). It was recognized through a high-throughput screen using low-passage primary GBM cells; it selectively targets GBM cells and exerts minimal harm to normal cells (Shi et al., 2019[18]). Gboxin accumulates in the mitochondria of GBM cells, exploiting their high mitochondrial membrane potential and elevated pH, leading to effective inhibition of GBM cell proliferation. However, the application of Gboxin in the treatment of GBM with TMZ-R is lacking.

In this study, we focused on whether Gboxin is effective against TMZ-R GBM cells. The experiments revealed that Gboxin suppresses the growth of TMZ-sensitive and TMZ-R GBM cells by promoting apoptosis and decreasing OXPHOS capacity and ATP production. This research reports a potential treatment strategy for TMZ-R GBM.

## Materials and Methods

### Cultivation of glioma cell lines and chemicals

The human LN229 and GBM8401 glioblastoma cell lines were maintained in Dulbecco's modified Eagle's medium (DMEM) supplemented with 2 % fetal bovine serum (FBS), penicillin and streptomycin. The cells were incubated at 37 °C in a 5 % CO_2_ atmosphere, as previously described (Tsai et al., 2018[24], 2019[25]). TMZ-R GBM8401 and LN229 sublines were created by collecting GBM cells that survived cultured in a high concentration as 500 μM of TMZ (Cat. No. T2577, Sigma-Aldrich) for 72 h weekly, as described previously (Tsai et al., 2019[25]). Normal human astrocytes, SVG p12, were grown in Eagle's minimum essential medium (MEM). The following chemical was used in this study: S-Gboxin (Cat. No. HY-111652, MedChemExpress).

### Cell viability

The OXPHOS inhibitor Gboxin was used to treat four different glioma cell lines, including resistant and nonresistant cells and control cells (SVGp12 normal human glial cells), and the CellTiter-96 AQ One Solution Cell Proliferation Assay (Promega, Madison, WI, USA) was used to assess cell proliferation. The cells were treated with specified concentrations of Gboxin for 24, 48, or 72 hours. After incubation, 20 μL of MTS solution (at a density of 10³ cells per well) was added to each well and incubated for 2 hours. The absorbance at 490 nm was measured via a Varioskan™ LUX multimode microplate reader (Thermo Fisher Scientific). Each experiment was performed three times independently.

### Flow cytometry analysis of the cell cycle and cell apoptosis

Annexin-V assays (#559763, BD Biosciences) were utilized to study cell apoptosis. The cells were grown in 6-well plates and subjected to Gboxin treatment for 48 h. The cells were collected and subjected to a procedure specified by the manufacturer. Finally, the percentage of apoptotic cells was assessed via flow cytometry (BD Biosciences).

To analyze the cell cycle, the cells were treated with Gboxin, stabilized in 70 % ethanol at 4 °C, stored at -20 °C overnight, and washed twice with chilled PBS. These cells were then dyed with a propidium iodide (PI) solution consisting of 50 μg/ml PI in PBS, 1 % Tween 20, and 10 μg/ml RNase A for a 30-minute period in the dark. Finally, the DNA content was assessed by assessing cell fluorescence via fluorescence-based cell sorting on a FACSCalibur flow cytometer (BD Biosciences).

### Cell lysate preparation and Western blotting

The cells were lysed in RIPA buffer (100 mM Tris-HCl, 150 mM NaCl, 0.1 % SDS, and 1 % Triton X-100) on ice for 10 minutes. Following lysis, the samples were centrifuged at 15000 rpm for 10 minutes, and the supernatant, containing the cell lysate, was collected. For protein analysis, 30 µg of lysate from each sample was loaded onto a 10 % SDS‒PAGE gel. The proteins were then transferred to polyvinylidene fluoride (PVDF) membranes (Millipore, MA, USA), which were incubated with 5 % skim milk in TBST for 1 hour at room temperature to block nonspecific binding.

Antibodies, including anti-MGMT (ab108630, Abcam), anti-survivin (ab76424, Abcam), anti-PLK2 (#14812S, Cell Signaling Technology), and anti-actinin (ACTN; sc-17829, Santa Cruz Biotechnology) antibodies, were used for Western blot analysis. The protein bands were detected by using enhanced chemiluminescence (ECL) detection reagent (GE Healthcare, Piscataway, NJ, USA) following the manufacturer's instructions. The band density was analyzed using ImageJ software (NIH Image, Bethesda, MD) and normalized to ACTN as indicated.

### Measurement of the oxygen consumption rate (OCR)

We assessed the oxidative respiration of glioma cells by using a Seahorse Bioscience XFp Analyzer and the Seahorse Cell Mito Stress Test Kit (Seahorse Bioscience, North Billerica, MA, USA). Briefly, glioma cells were seeded in a 10 cm culture dish and allowed to incubate overnight. The following day, the cells were treated with Gboxin as described earlier. After 48 hours of treatment, the cells were transferred to an XFp microplate and incubated in a seahorse XF DMEM Medium with a pH of 7.4 (#103575-100, Agilent Technologies). The OCR was then measured in the steady state; next oligomycin (1 μM), carbonyl cyanide 4-[trifluoro methoxy] phenylhydrazone (FCCP; 0.5 μM) and a mixture of rotenone (0.5 μM) and myxothiazol (1 μM) were sequentially added to the wells to determine the maximal and nonmitochondrial respiration rates.

### 10X Genomics single-cell RNA sequencing (scRNA-Seq) and analysis

LN229 TMZ-R cells treated with DMSO or Gboxin for 24 or 48 h were collected for scRNA-Seq via the GemCode^TM^ single-cell platform and Chromium^TM^ Single Cell 3' Reagent Kit v2 (10X Genomics, Pleasanton, CA, USA) according to the manufacturer's protocol. After the production of single-cell gel beads-in-emulsion, we performed reverse transcription (RT) with an Applied Biosystems^TM^ Veriti^TM^ 96-Well Thermal Cycler (Thermo Fisher Scientific, Foster City, CA, USA). SPRIselect beads (Invitrogen, Carlsbad, CA, USA) were used for purifying amplified cDNA, which was then subjected to sequencing on a NovaSeq platform (Illumina, San Diego, CA, USA).

The Cell Ranger software pipeline (version 2.0) provided by 10X Genomics was employed to analyze the scRNA-Seq data.

### Bioinformatics search

GENT2 provides prognostic data for a gene of interest. To analyze the associations between PLK2 expression and survival in different subtypes of glioma, we used the Gene Expression Database of Normal and Tumor Tissues 2 (GENT2) online tool (http://gent2.appex.kr/gent2/) (Park et al., 2019[15]). The relationship between PLK2 expression and the survival time of glioma patients was also examined using data from the Chinese Glioma Genome Atlas (CGGA) (http://www.cgga.org.cn/index.jsp) (Zhao et al., 2021[28]).

### Animal studies

All animal experiments were performed in accordance with the Institutional Animal Care and Use Committee of the National Defense Medical Center (IACUC-21-272). NOD. CB17-*Prkdc*^sicd^/JNarl (NOD SCID) mice were purchased from Lasco Co., Ltd. (Taiwan). The mice were housed in groups of four in the NDMC animal facility for the entire tumor generation period. For the subcutaneous xenograft model, 1 × 10^7^ LN229 TMZ-R cells in Matrigel^®^ were injected into the right flanks of 4-week-old male SCID mice. The tumor size was calculated using the following formula: (a+b)^2^/2, where "a" is the longest diameter of the tumor and "b" is the longest diameter perpendicular to "a". Once the tumor size exceeded 100 mm^3^, the mice were arbitrarily separated into 2 groups: a control group (n=7) and a treatment group receiving Gboxin (n=7). Gboxin (10 mg/kg) was administered by intraperitoneal injection (i.p.) once daily for a total of 4 weeks. The mice were sacrificed on day 49 post-tumor inoculation, and the subcutaneous tumors were harvested for weight measurement.

### Statistical analysis

All the experiments were conducted in triplicate, and the results are presented as the means ± standard deviations (SDs). Groups were compared via Student's t test, and differences were deemed statistically significant at a p value < 0.05.

## Results

### Gboxin suppresses the growth of GBM cells

**Figure 2:**
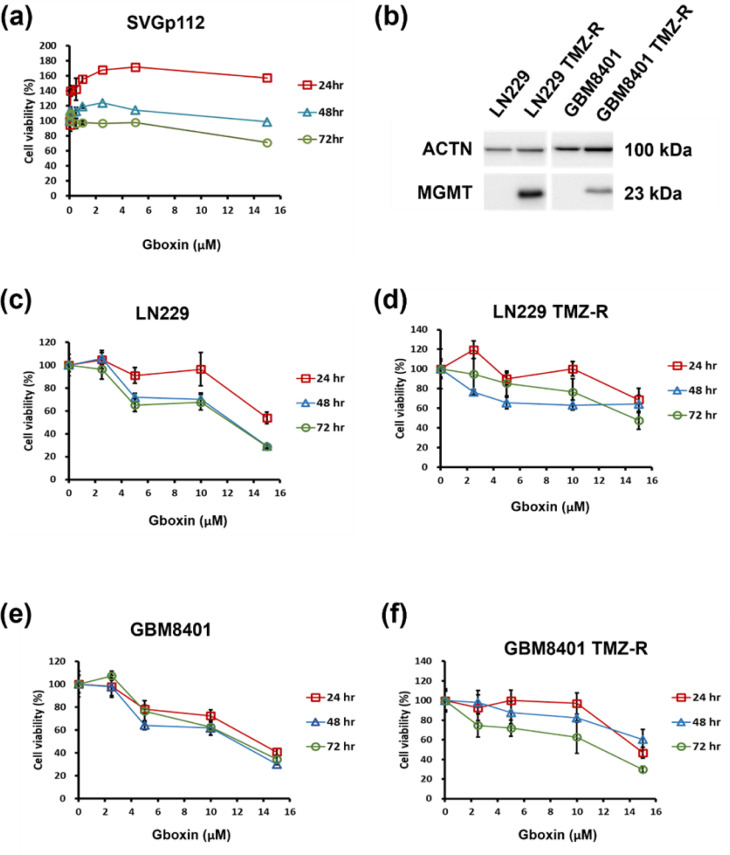
Cytotoxic effects of Gboxin on SVGp12 astrocyte cells and control and TMZ-R glioma cells. (a, c-f) Cell viability assays demonstrating the concentration-dependent response of SVG-p12, control, TMZ-resistant LN229, and GBM8401 cells to Gboxin treatment (0-15 μM) for 24, 48, and 72 h. Symbols depict the means ± S.D.s (n = 3). (b) MGMT protein levels were detected in parental and TMZ-R GBM8401 and LN229 cells. Whole-cell lysates were prepared and analyzed via Western blotting, with α-actinin used as the loading control. These results represent data from a single experiment.

To evaluate the repressive effect of Gboxin on normal astrocyte (control) and GBM cell growth, we used an MTS assay to assess the viability of SVGp12 cells (Figure 2a(Fig. 2)) and parental and TMZ-R glioma cells (Figure 2c-f(Fig. 2)) treated with the indicated concentrations of Gboxin for 24, 48, and 72 h. The TMZ-R phenotype of LN229 and GBM8401 cells was confirmed according to the expression of MGMT (Figure 2b(Fig. 2)). The percentage of viable GBM cells decreased in a concentration-dependent manner after treatment with Gboxin for 24, 48 or 72 h. The calculated half-maximal concentration (IC_50_) values of the parental LN229 and GBM8401 cells at 72 h were 12.77 μM and 12.23 μM, respectively, whereas they were 14.57 μM and 11.92 μM for the corresponding TMZ-R lines. Gboxin did not have a significant cytotoxic effect on SVGp12 calls except it is was applied at a high concentration. Moreover, low concentrations of Gboxin induced the growth of SVGp12 cells at 24 h (Figure 2a(Fig. 2)). These results demonstrate that Gboxin inhibits the growth of TMZ-sensitive and TMZ-R GBM cells and has no significant cytotoxic effect on normal controls (SVGp12 cells).

### Gboxin suppresses the proliferation of human GBM cells

**Figure 3:**
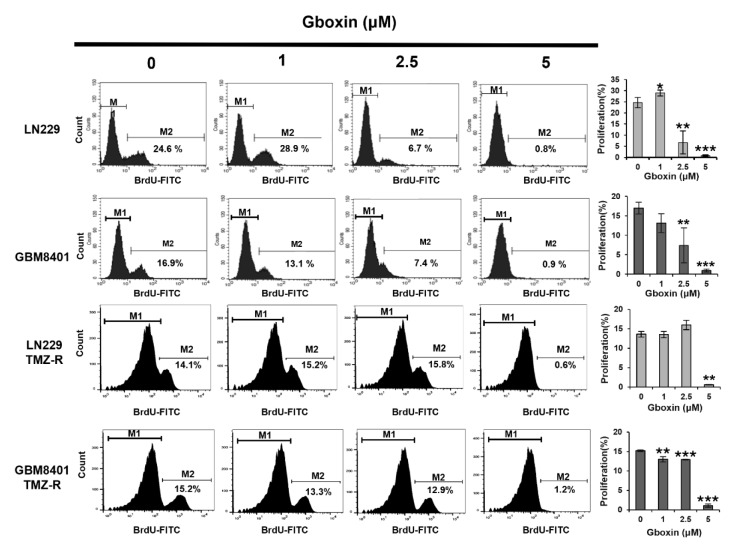
Gboxin dose-dependently suppresses the proliferation of GBM cells. The cells were incubated with Gboxin for 48 hours, stained with BrdU and analyzed via flow cytometry. M1 represents BrdU-negative cells, whereas M2 represents BrdU-positive cells. BrdU-positive cells indicate proliferative ability. The cells not treated with BrdU served as a blank control. The results are expressed as the mean ± S.D. (n = 3); *p<0.05, ** p < 0.01 and *** p < 0.001

To assess the influence of Gboxin on cell proliferation, we performed BrdU labeling and flow cytometry analysis. As shown in Figure 3(Fig. 3), Gboxin repressed proliferation in a concentration-dependent manner in both parental and TMZ-R LN229 and GBM8401 cells. The percentage of proliferating cells decreased from 24.6 % to 0.8 % in LN229 cells and 16.9 % to 0.9 % in GBM8401 cells but was 14.1 % to 0.6 % and 15.2 % to 1.2 % for the corresponding TMZ-R lines. However, LN229 and GBM cells appear to have different sensitivities to Gboxin, and the LN229-TMZ cells seem to be more resistant to the drug (2.5 µM) than the parental line. These results indicate that Gboxin suppresses the proliferation of GBM cells.

### Gboxin promotes apoptosis and downregulates antiapoptotic markers in TMZ-R cells

**Figure 4:**
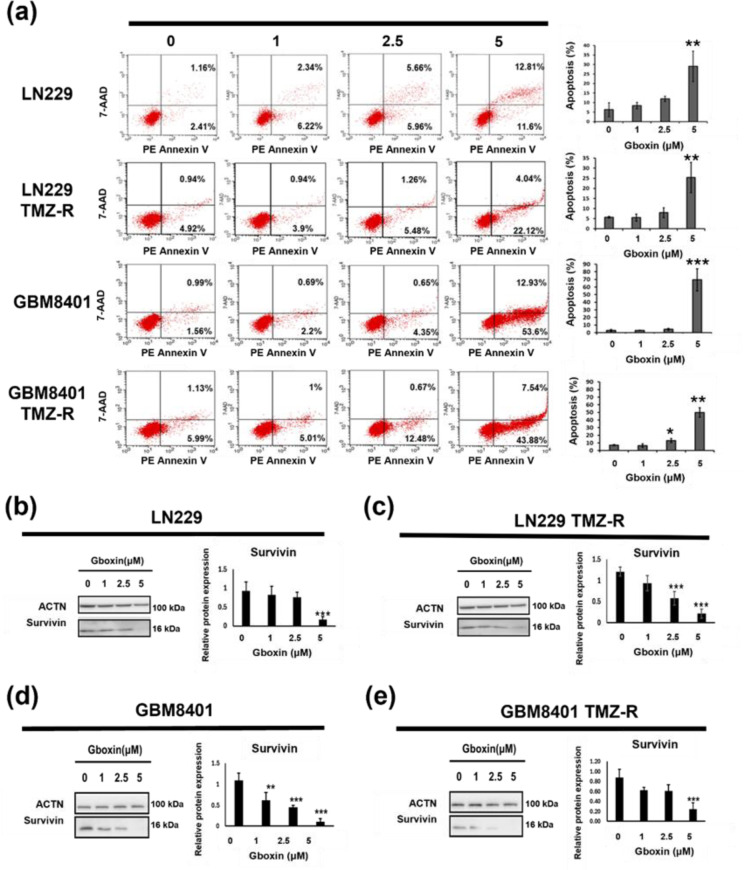
Gboxin dose-dependently induces apoptosis in GBM cells. (a) Apoptosis analysis of glioma cells treated with Gboxin for 48 h via 7-AAD/PE Annexin V staining. The apoptotic cells (the right lower quadrant represents early stages, and the right upper quadrant represents late stages) were identified via FCS and are represented as percentages. The results are presented as the mean ± S.D. (n = 3); * p < 0.05. (b-e) parental and TMZ-R LN229 and GBM8401 glioma cells were treated with specified concentrations of Gboxin for 48 hours. Whole-cell lysates were prepared and analyzed by Western blotting to assess survivin expression; ACTN was used as the loading control. This image is representative of data from three independent experiments. The results are shown as the means ± S.D.s (n = 3); ** p<0.01; *** p<0.001

Because a previous report revealed that the anti-GBM mechanism of Gboxin is associated with apoptosis, we utilized 7AAD and Annexin V cytometry to examine its influence on apoptosis. As shown in Figure 4a(Fig. 4)*, *Gboxin dose-dependently induced apoptosis in both parental and TMZ-R GBM cells, with an increase in the percentage of apoptotic cells from 3.57 % to 24.41 % in LN229 cells (*p<0.01*) and 2.55 % to 66.53 % in GBM8401 cells (*p<0.001*); however, in the corresponding TMZ-R lines, the percentage of apoptotic cells increased from 5.86 % to 26.16 % (*p<0.01*) and 7.12 % to 51.42 % (*p<0.01*), respectively. Overall, Gboxin induced more apoptosis in the GBM8401 cell compared to the LN229 cells in both sensitive and resistant lines. Next, we assessed the expression of survivin, an anti-apoptotic marker, in parental and TMZ-R LN229 and GBM8401 cells after Gboxin treatment via Western blotting. As shown in Figure 4b-e(Fig. 4), Gboxin inhibited the expression of survivin in a concentration-dependent manner. In summary, these findings indicate that Gboxin promotes the apoptosis of TMZ-sensitive and TMZ-R GBM cells.

### Gboxin treatment decreases the percentage of GBM cells in the G2/M phase

To determine whether alterations in the cell cycle distribution are involved in the reduction in cell viability, we assessed the effect of Gboxin on the cell cycle distribution via flow cytometry. Parental and TMZ-R LN229 and GBM8401 cells were treated with the indicated doses of Gboxin for 48 h.

**Figure 5:**
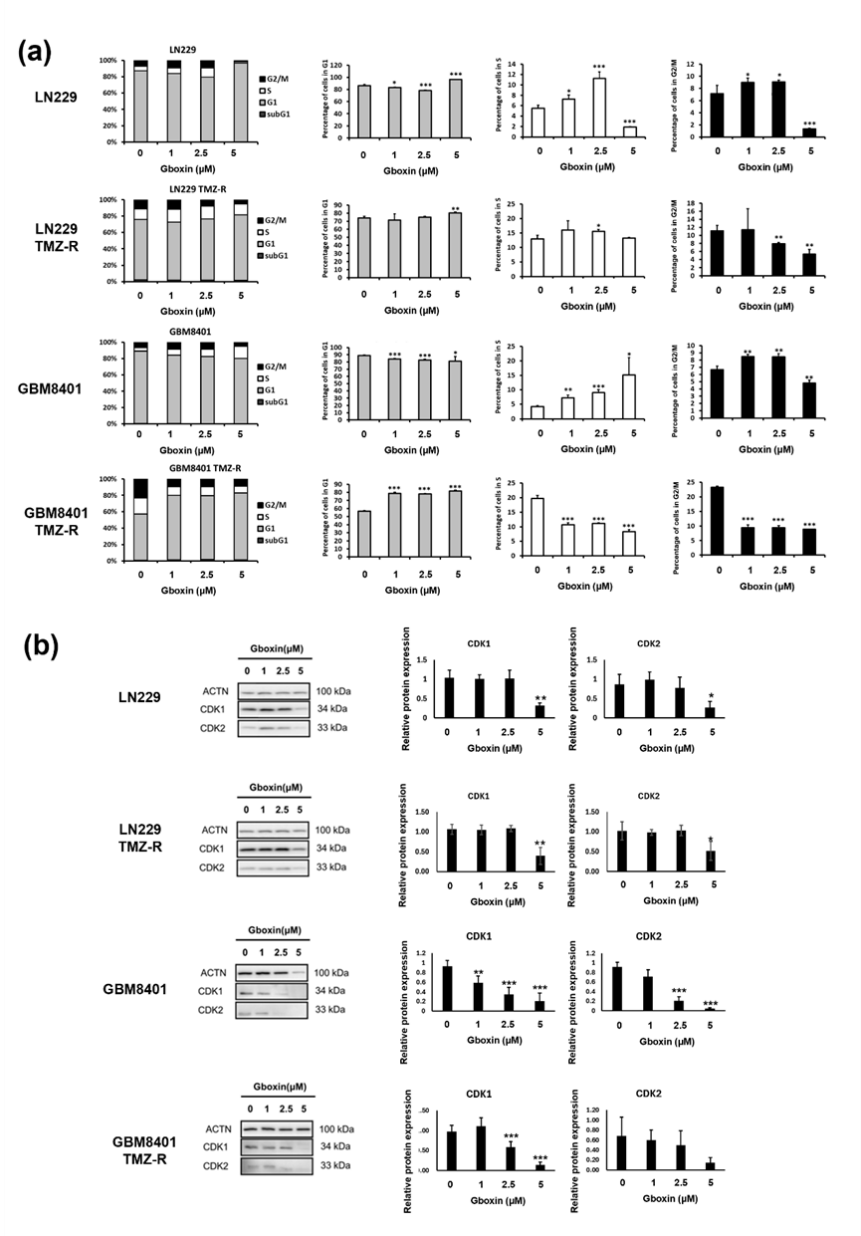
Gboxin arrests the cell cycle at the G1 or S phase in GBM cells. (a) Flow cytometry analysis of the cell cycle was performed following PI staining of control and Gboxin-treated parental and TMZ-R LN229 and GBM8401 cells. The ratios of cells in each phase were determined via CellQuest software (Becton Dickinson). The data are based on results from independent experiments (n≥2). * *p* < 0.05, ** *p <* 0.01, and *** *p* < 0.001 vs. controls according to Student's *t* test. (b) Analysis of CDK1 and CDK2 expression in parental and TMZ-R LN229 and GBM8401 cells treated with different doses of Gboxin. This image is representative of data from three independent experiments. The quantification of these levels of proteins relative to those of ACTN is displayed in the right panel (*n* = 3). * *p* < 0.05, ** *p <* 0.01, and *** *p* < 0.001 vs. control cells

The results revealed that Gboxin causes cell cycle arrest at the G1 phase in LN229, LN229 with TMZ-R, and GBM8401 with TMZ-R cells but induces arrest at the S phase in GBM8401 cells. Specifically, 86.3 % of the control LN229 cells were in the G1 phase, but after treatment with Gboxin, this percentage increased to 96.6 % (*p<0.001*). A similar effect was observed in LN229 TMZ-R cells treated with Gboxin, where 74.0 % of the control cells were in the G1 phase, and 80.2 % of the treated cells were in the G2 phase (*p<0.01*). Similarly, in GBM8401 TMZ-R cells treated with Gboxin, 56.7 % of the control cells were in the G1 phase, and 81.6 % of the treated cells were in the G1 phase (*p<0.001*). However, in parental GBM8401 cells treated with Gboxin, the percentage of cells in the G1 phase decreased from 89.26 % to 81.16 % (*p<0.05*), and the percentage of cells in the S phase increased from 4.1 % to 15.1 % (*p<0.05*). These data indicate that Gboxin arrests the GBM cell cycle at the G1 or S phase (Figure 5a(Fig. 5)). 

To further validate this observation, we probed for important proteins responsible for G1 or S phase arrest. We assessed the expression of the CDK1 and CDK2 proteins by Western blot, which both decreased in a dose-dependent manner (Figure 5b(Fig. 5)). Besides, CDK1 levels appeared to decrease more rapidly in the GBM8401 cells compared to the LN229 cell.

### Gboxin decreases the OXPHOS capacity and ATP production of GBM cells

**Figure 6:**
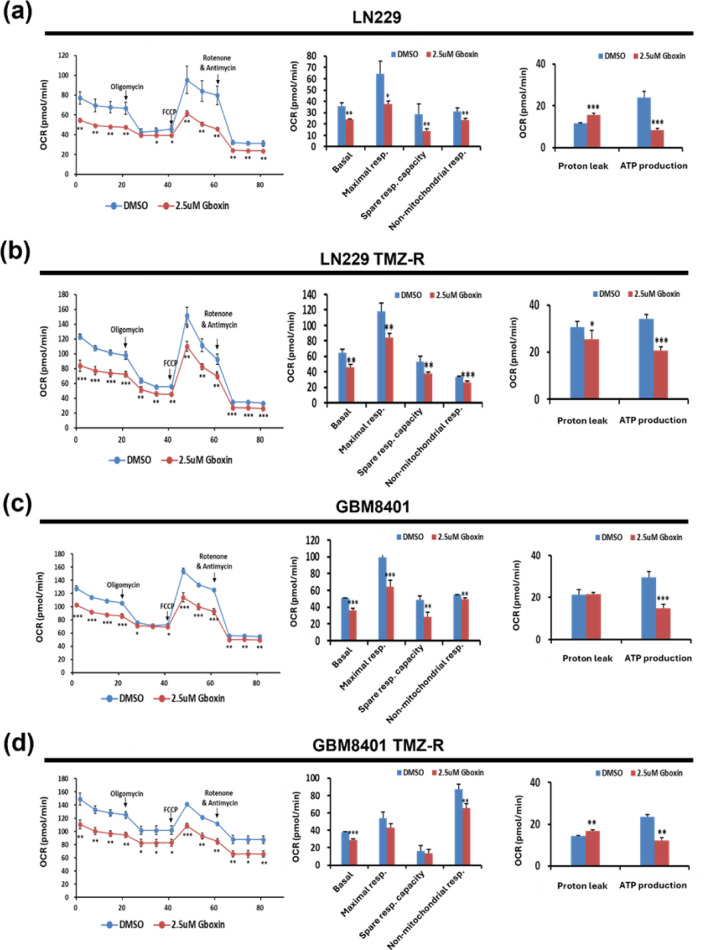
Gboxin inhibits mitochondrial OXPHOS in parental and TMZ-R GBM cells. Gboxin-treated glioma cells for 48 h were analyzed for bioenergetics via a Seahorse XFp Bioenergetic Flux Analyzer. The compounds, including oligomycin, FCCP, and rotenone/antimycin A, were sequentially added to measure key parameters of mitochondrial function. The oxygen consumption rates (OCRs) of both parental and TMZ-resistant LN229 (a, b) and GBM8401 (c, d) cells are presented in plots, highlighting the differences in basal respiration, maximal respiration, spare respiratory capacity, and nonmitochondrial respiration. The data are derived from separate experiments (n≥2). *p < 0.05; ** p < 0.01; *** p < 0.001

To investigate the effect of Gboxin on the energy metabolism of GBM cells, we used a Seahorse Bioanalyzer. Notably, reductions in OXPHOS-related factors, including the OCR, basal respiration rate, and ATP production, were noted in both parental and TMZ-R LN229 (Figure 6a, b(Fig. 6)) and GBM8401 (Figure 6c, d(Fig. 6)) cells incubated with Gboxin compared to those not treated with Gboxin. Interestingly, there are different effects on proton leak, with increases in LN229 and GBM8401 TMZ-R clones but a decrease in LN229 TMZ-R, and no change in GBM8401 clones. These results show that Gboxin alters the OCR and mitochondrial respiration. Hence, dysregulation of energy metabolism contributes to the anti-GBM effect of Gboxin.

### Single-cell RNA sequencing revealed heterogeneity among Gboxin-treated cells after 24 and 48 h

**Figure 7:**
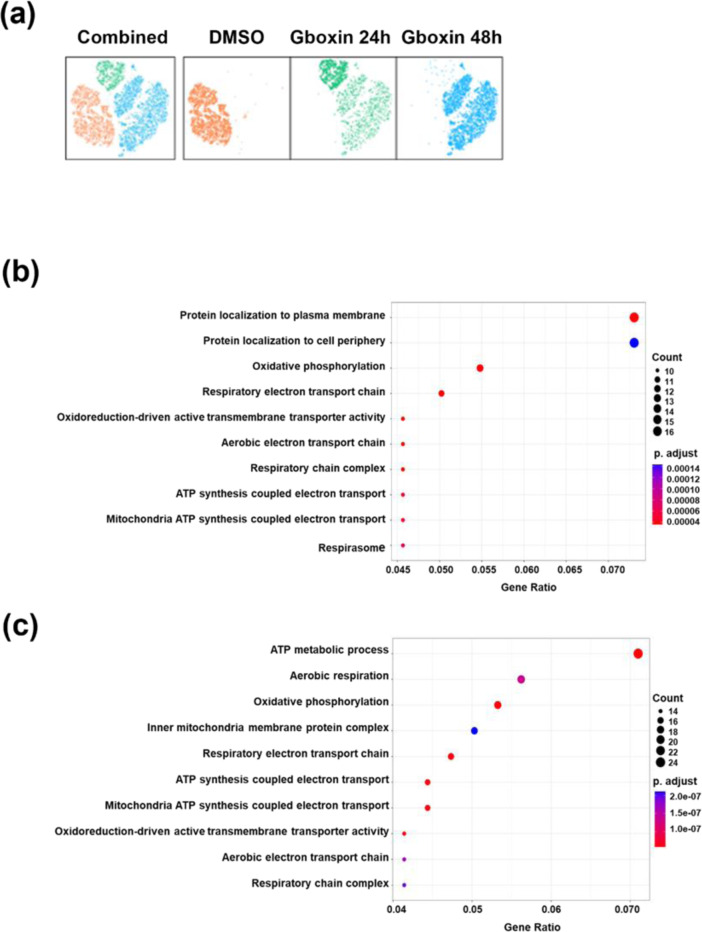
ScRNA-seq profiling and KEGG enrichment analysis of LN229 TMZ-R cells treated with the control and Gboxin (24 h or 48 h). (a) A t-SNE plot showing the distribution of three major cell types in LN229 TMZ-R cells following Gboxin treatment. The left figure combines all cell groups, including DMSO, 24 h, and 48 h Gboxin treatment. The orange color represents the DMSO control, while the green and blue colors represent Gboxin treatment for 24 hours and 48 hours, respectively. (b, c) Kyoto Encyclopedia of Genes and Genomes (KEGG) pathway analysis was performed on the downregulated genes at 24 h (b) and 48 h (c). In the resulting plot, the y-axis represents the KEGG pathway terms, whereas the x-axis represents the gene ratio, which indicates the proportion of enriched genes within a specific KEGG pathway relative to the total number of genes in the input list.

To characterize and distinguish cell-to-cell variation within a cell population at the transcriptome level, we performed scRNA-seq on LN229 TMZ-R cells treated with Gboxin for 24 and 48 h. As shown in Figure 7a(Fig. 7), the pathway enrichment scores of cell clusters significantly changed after Gboxin treatment at 24 h and 48 h compared to DMSO control. The 10 most altered pathways at 24 h and 48 h are shown in Figure 7b and 7c(Fig. 7). Most of the suppressed pathways were associated with energy metabolism, especially at 48 h; these pathways included the ATP metabolic processes, aerobic respiration, and OXPHOS. These results verify that the glioma-suppressing ability of Gboxin is related to the regulation of energy metabolism.

**Figure 8:**
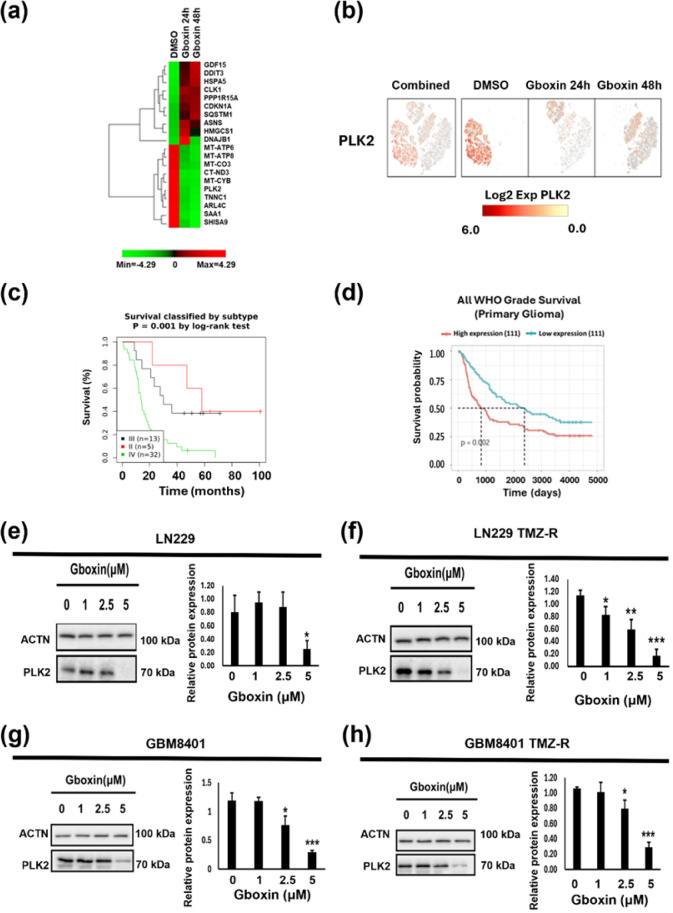
Identification of PLK2 as a potential downstream target. (a) Representative image of the top 10 upregulated and downregulated genes in LN229 TMZ-R cells treated with Gboxin for 48 h. (b) A t-SNE plot showing the distribution of PLK2 gene expression in LN229 TMZ-R cells treated with Gboxin for 24 or 48 h. (c, d) Kaplan-Meier survival analysis of glioma patients from the GENT2 (c) and CGGA (d) online platforms grouped based on the mRNA expression level of PLK2. (e-h) PLK2 protein expression in parental and TMZ-R GBM cells treated with specified doses of Gboxin for 48 h. Whole-cell lysates were collected and analyzed by Western blotting. This image is representative of data from three independent experiments. The quantification of these levels of proteins relative to those of ACTN is displayed in the right panel. The data are shown as the means ± SEMs (n = 3); *p < 0.05; ** p < 0.01; *** p < 0.001

We further confirmed the ten most up- and downregulated genes (Figure 8a(Fig. 8)) and analyzed the correlations of their expression levels with clinical features. After reviewing the literature, we selected PLK2 as a potential target. The proportion of cells expressing PLK2 among LN229 TMZ-R cells was significantly decreased after Gboxin treatment for 24 and 48 h (Figure 8b(Fig. 8)). By assessing data from the GENT2 and CGGA websites, we confirmed that PLK2 expression is significantly inversely associated with glioma patient survival (Figure 8c-d(Fig. 8)). Furthermore, Gboxin treatment suppressed the expression of the PLK2 protein in parental and TMZ-R GBM cells (Figure 8e-h(Fig. 8)). These results indicate that PLK2 is likely a downstream target of Gboxin.

### Gboxin attenuates TMZ resistance in a glioma subcutaneous xenograft model

**Figure 9:**
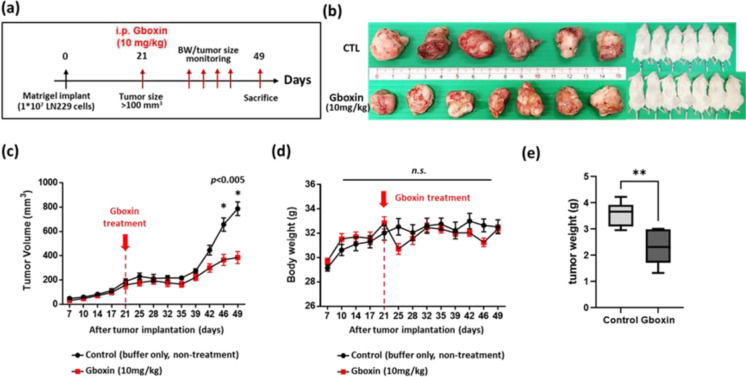
Gboxin inhibits TMZ-R-induced LN229 cell growth in a mouse subcutaneous xenograft model. (a) Experimental schedule for the TMZ-R LN229 subcutaneous xenograft model. (b) Photograph of the dissected xenograft tumors obtained from the right flank of the animals. (c, d) Quantitative analyses of the progression of tumor development (c) and body weight (d) of the animals. (e) Quantification of tumor weight in the control and treatment groups. * p < 0.05; ** p < 0.01

To assess whether Gboxin attenuates TMZ-R *in vivo*, we used a subcutaneous xenograft model (Figure 9a(Fig. 9)). A total of fourteen NOD SCID mice were subcutaneously injected with LN229 TMZ-R cells, but one mouse died the next day. Subcutaneous tumors formed over a period of 3 weeks. The samples were then randomly divided into two groups: the Gboxin treatment group (n=7) and the buffer control treatment group (n=6). The mice in each group were intraperitoneally administered either Gboxin or buffer at a dosage of 10 mg/kg/day for up to 4 weeks. Tumor growth was assessed during treatment. Compared with buffer treatment, Gboxin treatment resulted in significant inhibition of tumor growth compared to the control, in terms of tumor size, growth rate and tumor weight (3.57 g vs. 2.20 g, *p<0.001*) (Figure 9b-c, e(Fig. 9)). Moreover, there was no significant difference in mouse weight between the Gboxin treatment group and the control group (Figure 9d(Fig. 9)). These results indicate that Gboxin has potent repressive effects on TMZ-R-induced glioma growth* in vivo*.

## Discussion

The invasive behavior of GBM cells is influenced by changes in gene expression and interactions with the brain microenvironment. Tumor cells also undergo metabolic adaptation to the diverse conditions they encounter outside the tumor core, and these sites vary in nutrient and oxygen availability. Moreover, the acquisition of TMZ resistance results in GBM treatment failure. In this study, we aimed to determine whether Gboxin is a good treatment option for TMZ-R GBM. The experimental results demonstrated that Gboxin significantly suppressed TMZ-sensitive or TMZ-R GBM cell growth. The mechanisms include promotion of apoptosis and induction of G1-phase cell cycle arrest. Experiments in the subcutaneous xenograft model confirmed that Gboxin inhibits TMZ-R growth *in vivo*.

Shi et al. first confirmed that Gboxin suppressed GBM growth rather than normal astrocyte growth because GBM cells have impaired mitochondrial permeability transition pore function (Shi et al., 2019[18]). Thus, pH regulation in GBM cells is disrupted, resulting in the accumulation of Gboxin in the mitochondrial matrix. Metabolic rewiring can trigger epigenetic changes, posttranslational modifications, or alterations in metabolite availability (Ferrari et al., 2019[5]). Recent studies emphasize that these metabolic adaptations are crucial for cell survival and proliferation during invasion (Kreuzaler et al., 2020[9]). In this study, Gboxin had similar suppressive effects on metabolic features, including basal and maximal respiration and ATP production, in TMZ-sensitive and TMZ-R GBM cells (Figure 6(Fig. 6)). Besides, single-cell analysis also revealed that more than half of the 30 pathways related to ATP synthesis were associated with electron transport and ATP synthesis (Figure 7(Fig. 7)). To further elucidate the mechanism by which Gboxin affects TMZ-R, we measured the MGMT protein level. However, there was no significant change in TMZ-R GBM cells after Gboxin treatment (data not shown). These results indicate that metabolic rewiring is a potential strategy for TMZ-R GBM treatment. 

Targeting energy metabolism and disrupting the cell cycle are prominent mechanisms studied for anti-cancer strategies (Bai et al., 2023[3]). In this study, we revealed that Gboxin arrested TMZ-sensitive and TMZ-R GBM cells in the G1 or S phase by decreasing CDK1 and CDK2 expression in a dose-dependent manner at the protein level. These data indicate that inhibiting energy metabolism suppresses GBM cell growth, even in the context of drug resistance. Moreover, this metabolic stress often triggers cell death pathways, such as apoptosis or autophagy (Nikoletopoulou et al., 2013[11]). We found that Gboxin triggered an increase in the proportion of apoptotic cells, as shown by flow cytometry, and decreased survivin expression. Survivin is a suppressor of apoptosis and a promoter of cell division (Altieri, 2015[1]). Rivadeneira et al. reported that suppressing survivin suppresses OXPHOS in mitochondria, as evidenced by increased aggregation of detergent-insoluble complex II proteins and modestly diminished complex V amounts. Moreover, survivin knockdown decreases mitochondrial bioenergetics and decreases metastatic competency in prostate cancer PC3 cells (Rivadeneira et al., 2015[16]). Thus, these studies support that the suppression of survivin contributes to Gboxin-induced apoptosis.

PLK2 (polo-like kinase 2) belongs to the PLK (polo-like kinase) family, which affects DNA replication, the cell cycle (Zitouni et al., 2014[29]), and stress responses to DNA damage (Strebhardt, 2010[19]). Single-cell analysis revealed that PLK2 is a potential downstream target of Gboxin because of the significant downregulation of PLK2. The overexpression of PLK2 is correlated with poor survival in patients with colon rectal cancer (Ou et al., 2016[14]) or GBM (Ding et al., 2022[4]). Ou et al. reported that PLK2 triggers tumor growth and suppresses apoptosis in colorectal cancer (Ou et al., 2016[14]). Moreover, Ding et al. reported that PLK2 expression is associated with the prognosis of human GBM patients and that suppressing PLK2 has anti-GBM effects. Hence, our findings identify PLK2 as a prospective downstream target of Gboxin.

 This study demonstrated that the Gboxin effectively inhibited the growth of both TMZ-sensitive and TMZ-resistant glioma cell lines, with some differences observed between the cell lines. For example, LN229 TMZ-R cell line exhibited a low Seahorse proton pump leak, while GBM8401 showed no change, and LN229 and GBM8401 TMZ-R cell lines displayed an increase. Additionally, GBM8401 cell line was more sensitive to apoptosis compared to LN229 cell line. These findings align with previous research indicating variability in drug response among glioma cell lines (Tiek et al., 2018[23]). The observed differences may be attributed to inherent genetic variations and metabolic profiles of the cell lines (Ha et al., 2021[7]).

## Conclusion

In summary, we discovered that Gboxin may be effective against GBM cells with and without TMZ resistance and exerts dosage-dependent effects; Gboxin induces apoptosis, disruption of energy metabolism, and downregulation of PLK2. These results indicate that altering metabolism is a promising strategy for attenuating TMZ resistance, which may reveal new opportunities for GBM management.

## Declaration

### Authors' contributions

Chia-Kuang Tsai and Dueng-Yuan Hueng conceived and designed the study. Chia-Kuang Tsai, Chin-Yu Lin, Yung-Lung Chang, and Dueng-Yuan Hueng conducted the research and assisted with methodology development. Chia-Kuang Tsai and Dueng-Yuan Hueng wrote the first draft of this manuscript. Chia-Kuang Tsai, Chin-Yu Lin, Yung-Lung Chang, Fu-Chi Yang, Chung-Hsing Chou, Yu-Chian Huang, and Dueng-Yuan Hueng interpreted the data. All the authors were involved in revising the manuscript and provided their approval for the final version submitted. The corresponding author certifies that all listed authors meet the required authorship criteria and that no qualified individuals have been left out.

### Funding

This study was supported by grants from the National Science and Technology Council (NSTC 113-2321-B-016-005, NSTC 112-2314-B-016−036-MY2 and NSTC 111-2314-B-016-053-MY2), Tri-Service General Hospital (TSGH-E-112231, TSGH-C02-112031 and TSGH-C03-113039), Taichung Armed Forces General Hospital (TCAFGH-E-111042 and TCAFGH-E-112049, and TCAFGH-E-113045), and the Ministry of National Defense Medical Affairs Bureau (MND-MAB-D-112075, MND-MAB-D-113058, MND-MAB-D-113087). The funding source had no role in any process of our study.

### Competing interests

The authors declare that they have no competing interests.

### Data sharing

The data generated during the current study are available from the corresponding author (Dueng-Yuan Hueng) upon reasonable request.

### Using Artificial Intelligence (AI)

Authors used artificial intelligence (AI)-assisted technologies to check grammar errors.

## Figures and Tables

**Figure 1:**
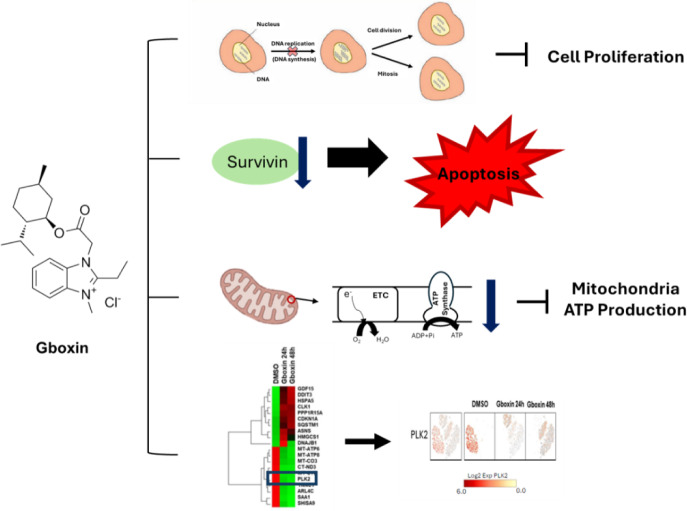
Graphical abstract
